# What determines voting behavior to host the Olympic games in the Rhine-Ruhr area: a multilevel model

**DOI:** 10.3389/fspor.2024.1372121

**Published:** 2024-05-01

**Authors:** John A. Menge, Christoph Bühren, Torsten Schlesinger

**Affiliations:** ^1^ Department of Sport Management, Ruhr University Bochum, Bochum, Germany; ^2^ Department of Human Locomotion, Chemnitz University of Technology, Chemnitz, Germany

**Keywords:** Olympic games, multilevel model, intangible effects, voting behavior, mega-event

## Abstract

This study analyzes factors influencing public support for hosting the Summer Olympics in the Rhine-Ruhr (RR) region in 2036 or 2040. Analyzing data from 14 municipalities, a multilevel model is used to account for individual and contextual factors. Despite a high overall endorsement rate of 67%, the study shows that consumer capital, pride, perception of sustainability, and historical significance significantly influence voting behavior. Surprisingly, structural factors at the municipal level do not show a significant influence. This research provides valuable insights for policymakers and organizers considering future Olympic bids in the RR region. The results emphasize the importance of effective communication to gain public support. This communication should highlight the environmental and economic sustainability of the host community, historical significance, and intangible benefits such as pride.

## 1 Introduction

Mega sports events, such as the Olympic Games (OG) and the FIFA Football World Cup, attract a vast number of spectators every four years—live at the sports venues and in front of the TV. With this large interest all over the world, interest in hosting these events has become more and more desirable for countries and policymakers (1). However, in the recent past, especially in Western countries, referenda on hosting the events were typically rejected (2), and the International Olympic Committee (IOC) requires the host nation to show that the population supports the event (3). Thus, before potential organizers start planning to give an application for OG, the expectations and attitudes in society must be checked. The DOSB (German Olympic Sports Confederation) has decided not to submit any application for OG in the future without a positive vote from the population as a binding mandate.

To the best of our knowledge, all research on individual voting behavior in the sports context has focused on personal factors, such as the inclination to vote in favor of subsidies for constructing new stadiums and arenas (4–6), the increase in political support through sport (7), and the voting behavior in a referendum on a mega-event (8, 9). Following the failed referenda in Germany for the 2018 and 2022 Winter OG in Munich and the 2024 Summer OG in Hamburg, Menge et al. (10) assessed the willingness to pay for the potential hosting of the 2036 OG within the population of the Rhine-Ruhr (RR) region. They found that 61% of the population of RR was in favor of the potential hosting, 31% against it, and 8% did not know whether to support a potential bid. However, public support in surveys before the referendum does not necessarily lead to a positive result in the actual referendum. For the Hamburg 2024 OG, a representative survey estimated a support rate of 56% for hosting the Games, but the result was only 48.4% in favor of the OG (11). In Innsbruck-Tyrol, the referendum towards the potential hosting of the Winter Olympics narrowly failed. Wicker and Frick (12) show that the environmental aspect played a crucial role in voter turnout: People who supported the green party and people who lived in communities affected by industrial emissions indicated lower levels of support. Feilhauer et al.'s (13) findings suggest that a stronger acknowledgment of the OA20 could have led to a positive voting outcome for the referendum in Tyrol—especially by reducing the expected financial burden, infrastructure costs and, above all, corruption, as well as strengthening the intangible effects, such as the population's trust in the IOC. With regard to the 2014 FIFA World Cup in Brazil, Oliveira Santos et al. (14) demonstrate that public's perception is a decisive factor for the residents' approval to host such an event. Therefore, transparency should be an important factor in the perception of the population. A referendum and transparent communication by the organizer and political decision-makers are consequently a crucial tool for hosting a major sporting event.

## 2 Rhine-Ruhr region as the potential host of the Olympics

In 2016, the privately funded “Rhein Ruhr City” (RRC) initiative proposed a concept in which the RR area would co-host the 2032 Olympics. This concept builds on the Olympic Agenda 2020^1^ that demands the reduction of the cost of hosting the Games. It was launched by the IOC since the costs for hosting skyrocketed in the last decades, leading to a decline in bids from host cities (15). Research on the OA20 has been conducted concerning the 2026 Winter Olympics. Schnitzer and Haizinger (16) analyzed official IOC documents and feasibility studies of the bidders for this event to investigate whether the OA20 plays a decisive role for the future of the OG. Furthermore, they conducted expert interviews and concluded that the future Olympic legacy may be less focused on building iconic infrastructure but more on trying to fulfill the city's long-term strategies. Using meta-organizational theory and 35 semi-structured interviews, Bazzanella et al. (17) point out that the implementation of the OA20 can lead to opportunities. For instance, the host regions could benefit from various shared expertise and knowledge of Milan and Cortina d'Ampezzo, which may contribute to the successful realization of the 2026 Olympic Winter Games. However, it can also lead to challenges, as the organizers and the population may have different motivations and objectives. This is of particular interest to policymakers and RR organizers because of the opportunities and challenges of such a multi-city hosting. As mentioned in the introduction, the implementation of the OA20 for the referendum in Tyrol for the 2026 Winter Olympics could hypothetically have led to a positive result of over 50% approval (13).

In the RR area, high-level sports infrastructure already exists. However, the IOC named Brisbane 11 years in advance of the event as the host for the OG 2032. Nevertheless, policymakers and RRC still discuss the idea of RR as a possible host for the OG 2036 or later. To analyze the level of public support, conducting a referendum would be a critical step toward assessing the viability of continued planning efforts.

The concept of RRC is to unite 14 different cities as a collective host region. While in the past certain competitions were already held in different cities, there was always a single host city that served as the official naming partner of the Games. There are many advantages of hosting the OG as a region, including the presence of pre-existing sporting facilities, well-developed transportation systems, and hotel accommodations. All this may reduce the cost of hosting significantly. Additionally, the likelihood of subsequent expenses is probably reduced, such as the emergence of “white elephants”—sports facilities that are not maintained or utilized after construction. Moreover, the dense population in RR would benefit from modernized transport infrastructure after the event. On the organizational level, the different host cities can also benefit from each other's knowledge and expertise (17). These are just a few benefits following the hosting of the OG that proponents of the OG regularly claim. The problem is that most of these benefits have generally been refuted by scholars (15). However, hosting as a region may increase the likelihood of these benefits being realized.

Despite the advantages of hosting the OG as a region, the people of the different cities involved may have various perspectives regarding these benefits and potential drawbacks of hosting the event. This is related to the fact since cities/regions can be developed differently, some regions have more problems with the infrastructure than others, and the social circumstances can also be different—thus, the regions are also likely to benefit differently from major sporting events. Therefore, contextual factors that differ by cities in the region, such as political or economic conditions, should be considered when estimating the potential support of hosting the OG in a region.

## 3 The need to consider the context → Why does the context matter?

**Figure 1:**
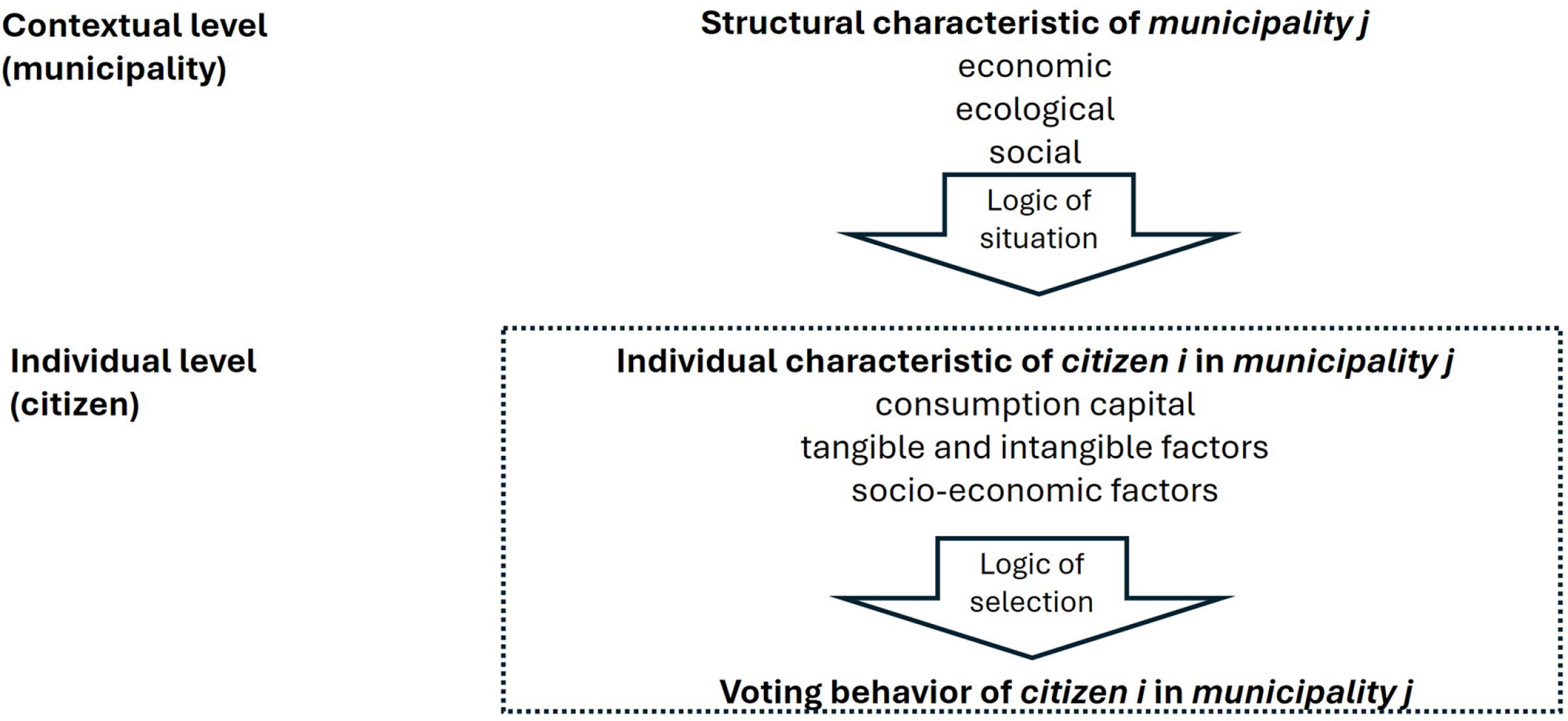
Multilevel framework to explain the voting behavior of citizens regarding the hosting of the OG in the municipality (own figure based on 23).

In the context of sports, multilevel model (MLM) studies have been conducted on the factors of sport participation (18–20) and member engagement in voluntary sports clubs (21–23). All studies find an influence of contextual characteristics. The need to consider the social context in relation to the hosting of OG in RR can be underlined by (a) methodological and (b) theoretical-conceptual arguments:

(a) In RR's approach, 14 different host communities are involved in the potential hosting of the OG. Thus, their citizens are directly affected by the impact of the OG. Therefore, an exclusive consideration of individual characteristics seems insufficient to explain the variation in citizens' attitudes towards the OG and their voting behavior. Attitudes are likely shaped by both individual characteristics and the social context of the municipalities. This is because the citizens of a municipality are subject to common situational influences and structural conditions that are unique to that municipality, and these characteristics distinguish them from citizens of other municipalities. Accordingly, explanatory models that consider both individual and context-specific characteristics are likely to provide a more accurate picture of social reality than models that only consider either individual or contextual aspects.
(b) Multilevel analyses are necessary from a statistical point of view to evaluate hierarchically structured data sets. Hierarchical data structures exist when data can be grouped (“nested”)—i.e., individual study units at the lower level can be clearly assigned to the higher level (here: citizens to municipalities)—and the higher level is composed exclusively of elements from the lower level (24). In our study, this means that in analyses of citizens' attitudes and voting behavior, the measured values are not completely independent of each other, as citizens can be clearly assigned to a municipality. Consequently, citizens' attitudes and voting behavior are not only related to individual data (analysis of differences between individuals) but also to corresponding structural data (analysis of differences between groups) (see Figure 1). In hierarchical data structures, the individual observations at level 1 (citizen level) are not independent of each other but are subject to common contextual influences (municipality level) that can lead to similar attitudes and actions of the individuals within the contextual unit. The statistical model must acknowledge the hierarchical data structure so that the effect of individual and contextual characteristics can be adequately determined. The problems often associated with regression analysis, when dealing with hierarchical data structures, can be overcome with MLM. Basically, MLM analysis considers the hierarchical structure of different levels of data as a detailed representation of a complex reality. It makes theoretical considerations about the effects of level-specific variables accessible to an adequate empirical analysis (25). According to its basic principle, the MLM analysis removes the limitations of one level of analysis and ensures more precise and robust results with simultaneous estimates at different levels of analysis (individual and contextual).

This leads to the following research question: What individual and contextual factors influence the voting behavior of the people in the Rhine-Ruhr area regarding the possible hosting of the Summer Olympics in Rhine-Ruhr in 2036 or 2040? Hence, this study contributes to the research by analyzing the structural impact of the cities in RR on the support for hosting the OG in 2036.

## 4 Influences on voting behavior

### 4.1 Contextual factors

The rational choice model (26), commonly applied in the context of voting behavior (27), can explain the voting patterns observed among the local population. According to this model, individuals are viewed as rational actors who make choices based on their self-interest. Consequently, they are expected to vote for the party or candidate that will maximize their utility. This decision is not only derived from the individual characteristics of resident *i* (individual level) but also from the social environment of municipality *j* where the individual is living in and socially embedded (contextual level) (Figure 1). The logic of the situation in which the individual decision is usually made must therefore be used as a foundation for understanding that extends the concept of rational choice (28). Thus, the participating municipalities, with their specific economic, social, and ecological circumstances, represent encouragements or discouragements (logic of the situation), which can influence voting patterns according to individual preferences (logic of selection). We extend the rational choice model by including behavioral economic determinants such as consumption capital constraints, tangible and intangible effects, and socio-economic factors. We discuss these determinants in the next subsection.

Considering the voting behavior on hosting a mega-event, the contextual level consists of the communities' structural characteristics. In RR, each city has its specific social (e.g., identification or political distribution) and economic situation (e.g., debts per capita or tourists per capita) that are likely to affect individuals' behaviors and actions. Citizens evaluate hosting a mega-event either as an added value or a burden for the economic and social situation in their municipality. The structural characteristics that influence this evaluation are constant within one municipality and can vary between different municipalities.

### 4.2 Individual determinants

First, by repetitively engaging in the consumption of a specific good, such as actively and passively participating in sports, individuals can accumulate consumption capital (29). This implies that individuals perceive extra-utility by repeatedly experiencing events like OG or sports in general, which enables them to make better evaluations of these events. Individuals who regularly participate in sports, attend or watch sports competitions, and follow events like OG or the FIFA World Cup, are likely to have a preferred team or nation they support, comprehend the rules, and be familiar with the “superstars” (30). Thus, consumption capital could induce preferences in favor of hosting a mega-event (31, 32). In addition, factors such as pride or identification with the region must be taken into account when hosting major sporting events. This implies an extension of behavioral economics, as the basic assumption of neoclassical rationality of invariant preferences is softened (33). Nevertheless, the goal is still to optimize the utility for the individual (34).

Second, the impact of hosting mega-events on individual utility is influenced by tangible economic factors and intangible factors, which consequently influence individuals' voting behavior (30). These range from an increase in tourism and a boost in GDP on the one hand, and pride, prestige, and recognition of the city in the world on the other hand (1). However, next to the wide range of positive tangible and intangible factors, the events come with several negative externalities (1, 35). Specifically, the cost of hosting is typically discussed (36), as is pollution as a consequence of increased traffic (37, 38). Other negative externalities include crime and price inflation (39).

Third, individuals in different socio-economic environments are assumed to derive different levels of benefit from hosting mega-events (31, 40). Therefore, socio-economic factors, such as human capital, age, gender, or income, may shape individuals’ utility (30). Previous research has shown that socioeconomic factors are associated with the individual experience of hosting a mega-event (38). For instance, Streicher et al. (41) found that males and younger people were more likely to support the hosting of the Olympics in the US and Europe. However, this finding could not be confirmed by the review of Orlowski and Wicker (38). These results highlight how socioeconomic factors can differ in various settings and must therefore be considered as controlled factors.

## 5 Methods

### 5.1 Data collection and sample

In all 14 potential host municipalities, the influences of the individual and structural variables on the votes in favor or against hosting the OG 2036 in RR were analyzed (see Table 1 for an overview of the variables). Individual data were collected via an online survey after the Tokyo OG 2020, ensuring that participants memorized the event (31). The survey was distributed in several Facebook groups with content related to the host municipalities, an online newspaper in Moenchengladbach, and the social media channels of Bonn and Dortmund.

**Table 1:** Overview of variables and descriptive statistics.

Variable	Description	Mean	SD
Dependent variable			
Support	Share of supportive votes (1 = “Yes”)	0.668	0.47
Variables on individual level			
Club	Member of a sports club (1 = “Yes”)	0.48	0.5
Pride	Pride of hosting the Olympic Games (from 1 = “Do not agree at all” to 5 = “Fully agree”)	3.36	1.63
Pollution	Increase in pollution (from 1 = “Do not agree at all” to 5 = “Fully agree”)	2.84	1.29
Sustainable	Metropolitan region as a sustainable host (from 1 = “Do not agree at all” to 5 = “Fully agree”)	3.77	1.25
Nazi-Games	100 years after the “Nazi-Games” in 1936 (from 1 = “totally critical” to 5 = “as a clear opportunity”)	3.64	1.49
Age	Age in years	41.31	12.62
Sex	Sex (1 = “female”)	0.41	0.49
Education	Educational level in %		
	low education - below university entrance degree	17.31	
	medium education - university entrance degree	32.17	
	high education - university degree	49.42	
	no information	1.09	
Variables on structural level			
Place	Place attachment of residence (from 1 = “Does not apply at all” to 5 = “Fully applies”)	4.08	0.98
Green	Percentage of green votes in the last municipal election in 2020	22.3	5.69
CDU	Percentage of votes for the conservative party in the last municipal election in 2020	27	5.23
Tourist	Tourism overnight stays per capita in the municipalities in 2019 due to the corona pandemic	3.03	2.03
Debt	Per capita debt in thousands of each municipality in 2021	4.7	1.66

2,534 individuals started the survey and 1,848 finished it. Responses from individuals who did not live in any of the 14 municipalities (identified by postal code), were below 16 years of age, answered the question of supporting the hosting with “don't know yet” (<8%), or identified their gender as “non-binary” (.2%) were removed. After data cleaning, the final sample size was 1,467, which consisted of 59% males with an average age of 41.

Around 49% of the participants reported having higher education, and 39% had a medium monthly net income. The highest number of respondents came from Bonn (14%) and Dortmund (10%), whereas Recklinghausen (5%) and Krefeld (5%) had the lowest number of responses (Table 2).

**Table 2:** Overview of the distribution of the respondents across cities and their level of support for the potential hosting of the OG 2036 in RR.

Place of residence	Distribution		Support (%)	
	Absolut	%	Yes	No
“Aachen”	94	6.4	58.51	41.49
“Bochum”	109	7.4	68.81	31.19
“Bonn”	207	14.1	56.04	43.96
“Dortmund”	149	10.2	71.81	28.19
“Duesseldorf”	81	5.5	72.84	27.16
“Duisburg”	112	7.6	68.75	31.25
“Essen”	77	5.2	74.03	25.97
“Gelsenkirchen”	96	6.5	87.5	12.5
“Koeln”	112	7.6	62.5	37.5
“Krefeld”	69	4.7	68.12	31.88
“Leverkusen”	84	5.7	67.86	32.14
“Moenchengladbach”	111	7.6	63.06	36.94
“Oberhausen”	98	6.7	57.14	42.86
"Recklinghausen”	68	4.6	73.53	26.47

### 5.2 Measures

The binary variable of whether the participants support the hosting of the OG or not (*Support*) served as the dependent variable. The proxy for consumption capital was membership in a club (*Club*). Additionally, the survey included questions about the respondent's sense of pride regarding the hosting (*Pride*) as a positive intangible factor and the anticipated rise in pollution due to increased traffic as a negative intangible factor (*Pollution*). As 90% of the necessary infrastructure for hosting the OG already exists in RR, the respondents were asked if hosting the OG in RR is sustainable (*Sustainable*). In light of former DOSB President Alfons Hörmann's concerns that Germany should not apply to host the Olympics in 2036, 100 years after the Nazi-Games (42), respondents were asked whether they agree with his concerns or believe that it presents an opportunity to show the world a new image of Germany (*Nazi-Games*). Furthermore, we included individual socioeconomic characteristics, such as age (*Age*), gender (*Sex*), human capital (*Education*), and personal net income (*Income*). We also measured individual place attachment, but aggregated it for each municipality to analyze the structural variable (*Place*). This average place attachment in a municipality is one compressed factor (*α* = .91) obtained from the Abbreviated Place Attachment Scale (APAS) of Boley et al. (43).

Furthermore, we included political preferences, the role of tourists, and the financial situation in each municipality as structural variables. Previous studies generally observed that individuals who support the green party are less likely to favor the hosting of OG, while those who voted for the conservative party tend to support the hosting (2, 12). We used the percentage of individuals who voted for the green (*Green*) or conservative party (*CDU—Christian Democrats*) in the last municipal elections as control variables for the political influences of each municipality. The number of tourists per capita (*Tourists*) served as a control for crowding-out effects. These may appear when a mega-event discourages ordinary tourists or business travelers from choosing the host destination (15). Finally, we considered the financial problems of a municipality by its per capita debt (*Debt*).

### 5.3 Data analysis

The hierarchical nature of the data implies that individual factors (lower level) are nested within a contextual structure (higher level). This violates the assumption of independent residuals in an Ordinary Least Squares (OLS) model (25). The use of MLM enables the analysis of hierarchical data in an appropriate manner. It considers the effects between variables from multiple levels and may identify relationships that would remain undetected in OLS (44). Two conditions should be fulfilled in MLM: First, the theoretical assumption of a contextual-level influence should be reasonable. Second, the structure of the data should fit in the MLM analysis. As argued above, the theoretical influence of the contextual level—the structural variables in the municipalities—is given in our study. Next, we discuss what criteria must be met for MLM to make sense.

Previous literature struggled to agree on the criteria for using MLM (45). MLMs require a rather large sample size, where the number of different clusters is more important than the individual observations within the clusters (46). However, in several simulation studies, MLMs have been used with small clusters, such as 10 or fewer (47). Only 14 municipalities are hosting the OG; therefore, the cluster size is fixed in this study. Another indication for using MLMs is a high intraclass correlation coefficient (ICC). ICC represents the amount of variance attributable to the higher level—how much variance can be explained by the structural conditions in the estimation (44). Closely connected to the ICC is the design effect, which indicates to what extent the standard errors in a complex sample are underestimated compared to a simple random sample (46). It can be estimated using the average cluster size and the ICC (48). A large ICC (.9) with a low cluster size (2) results in the same result (design effect = 1.9) as a small ICC (.018) with a large cluster size (50) (49). Subsequently, the design effect is not solely determined by the ICC (45). For a general guideline, the design effect should be more than 2 to indicate the need for a multilevel model (50).

Our MLM consists of two steps. First, the null model (random intercept only model) is estimated, which solely consists of the regression intercept and examines how the total variance of the dependent variable is divided between the individual and structural levels. Second, the random intercept model is used. Its basic idea is that the intercept is different for each structural unit, but the slope coefficients are fixed and the same for all units. It includes all the individual and structural independent variables.

Useful measures of the model fit are the deviance, Akaike's information criterion (AIC), and the Bayesian information criterion (BIC). The deviance is the log-likelihood value of the model multiplied by −2 and serves as a comparison tool for different models. The closer the deviance is to 0, the better the model fit (44). However, it decreases automatically when more variables are included. Therefore, AIC and BIC should be considered when comparing models with different numbers of dependent variables. Similar to the deviance, the model fit is better when AIC and BIC are closer to 0. The variable *Income* has been excluded from the estimation due to a reduction in model fit.

We use the restricted maximum likelihood procedure for the estimations and report the Average Marginal Effects (AME) to facilitate the interpretation of the results (51). AME represents the average change in the dependent variable that is associated with a one-unit increase in the independent variable, holding all other variables constant at their mean values (52). Multicollinearity was not a problem (VIF <5).

## 6 Results

### 6.1 Descriptive statistics

Table 1 provides descriptive statistics. 67% of the respondents support the idea of hosting the OG in the metropolitan region of RR. The varying levels of support in the municipalities in RR are shown in Table 2, which suggests the influence of the structural factors on voting decisions and indicates the use of MLM.

On the individual level, 48% of the respondents have a club membership, and hosting the event would make them rather proud (on average, 3.36). They evaluate RR as a sustainable host due to the high number of already-built sporting infrastructure (3.77). 67% of the respondents regard hosting the event 100 years after the Nazi-Games as a chance to show a different picture of Germany, and 26% see this as critical. The average level of concern regarding pollution resulting from the event is not particularly high (2.84).

#### 6.1.1 Variables on structural level

In all 14 municipalities, residents generally have a strong sense of attachment to their city (4.08). The green party received an average of 22.2% of the votes, while the conservative party received an average of 27%. However, there were noticeable differences between the 14 municipalities. On average, each municipality hosted 3.03 overnight tourist stays per capita. The average per capita debt was 4.7 thousand euros, with significant variations among the 14 municipalities as well.

### 6.2 Multivariate analysis

Table 3 reports the results of the MLM with the binary dependent variable *Support*. First, the null model (Model 0 in Table 3) analyzes the share of the variance in the dependent variable that can be attributed to individual characteristics and contextual factors by estimating the ICC (.031). This means that 3.1% of the variance is explained by the differences between the 14 municipalities. Hayes (53) argues that an MLM is not necessary when the ICC is too low (<.05). However, Huang (45) replies that even an ICC of .01 may increase the Type I error rate in OLS models up to four times. Simulation studies indicate that even with low ICCs, the standard errors and confidence intervals of MLMs are relatively small (<10) when the models are kept relatively simple and straightforward (54). Therefore, in this analysis, we focus on the random intercept only and random intercept models (23). Furthermore, our design effect is above 4 and fulfills the “golden rule”, which states that it should be above 2 to require MLMs (46). Finally, according to Huang (45) and Tausendpfund (44), the use of MLMs should be based on theoretical arguments, such as the uniqueness of each municipality with its individual social and economic characteristics (28).

**Table 3:** Multilevel model with *Support* as the dependent variable.

		M0: Random intercept only	M1: Random intercept
Fixed part	Intercept	0.756***	−18.854***
Individual level			
Club (reference = yes)			
	No		−0.020**
Pride			0.032***
Pollution			−0.009**
Sustainable			0.020***
Nazi-games			0.036***
Sex (reference = male)			
	Female		0.016*
Age			−0.000
Education (reference = high education)			
	No Information		−0.034
	Low education		0.008
	Medium education		0.003
Structural level			
Green			0.000
CDU			0.000
Tourists			0.002
Debt			−0.000
Place			0.020
Random part			
Variance			
Individual level		3.282	3.219
Structural level		0.105	0.059
ICC		0.031	0.018
Deviance (−2 * log likelihood)		1,848.3	186.1
AIC		1,852.3	220.1
BIC		1,862.9	310.1
*N*			
Individual level			1,467
City		14	14

Coefficients are presented as average marginal effects.

* *p* < 0.05.

** *p* < 0.01.

*** *p* < 0.001.

Second, the random intercept model (Model 1 in Table 3) analyzes the support at the individual level controlling for structural differences. Except for age and human capital, all individual variables were significant. Having a club membership increased the support level by 2 percentage points. This proxy for consumption capital suggests that being a member of a club and regularly doing sports increases the utility derived from such an event. Both the positive and negative intangible proxies were significant in the expected direction: Being proud of hosting the OG increased its support by 3.2 percentage points, whereas expecting the OG to increase pollution reduced the support by 0.9 percentage points. The concept of hosting as a municipality, and therefore increasing the economic and ecologic sustainability, increased the level of support by 2 percentage points. Seeing the OG 2036 rather as a chance to illustrate a different Germany from the one of 100 years ago increased the level of support by 3.6 percentage points. Interestingly, women were 1.6 percentage points more likely to vote for the hosting.

Looking at the structural level, we find no direct significant influences. Neither the proportion of green nor conservative votes in the community significantly influenced the decision to vote for or against the hosting. Furthermore, the economic situation of the municipality seems to have no influence on voting behavior. Sports and the hosting of sporting events seem to be equally popular in all economic strata. Lastly, the aggregated place identity has no significant influence either.

## 7 Discussion, limitations, and future research

### 7.1 Discussion

This study considered the hierarchical data structure analyzing public support for the potential hosting of the OG 2036 in the RR metropolitan area. Furthermore, multilevel modeling was applied to incorporate structural factors of the 14 potential host communities.

About 67% of respondents were in favor of hosting the OG. In Atkinson et al. (40), 47% of the respondents in London were strongly supportive of the London 2012 bid, and an additional 32% were supportive. Wicker and Coates (9) observed a support level of 41.2% before the Hamburg 2024 referendum, and Wicker and Frick (12) observed a share of 53% of yes-votes in the referendum towards to potential hosting of the Winter Olympics 2026 in Innsbruck-Tyrol. The referenda on the possible hosting of the OG in Hamburg 2024 and Innsbruck-Tyrol 2026 narrowly failed. Notably, in the Innsbruck-Tyrol referendum, larger communities—such as Innsbruck—voted against the hosting. We observed a higher support level than Wicker and Coates (9) and Wicker and Frick (12), suggesting an already positive attitude, possibly due to the implementation of OG20 (13), towards the potential hosting of the OG in RR similar to Atkinson et al. (40).

Consumption capital plays a significant role in creating added value for the people of the hosting municipalities. People who are members of sports clubs are significantly more likely to support the hosting. Furthermore, respondents who would be proud of the potential hosting are more likely to cast a positive vote for the hosting. In line with Kurscheidt and Prüschenk (36), Wicker and Coates (9), Feilhauer et al. (13), and Wicker and Frick (12), the idea of an ecologically and economically sustainable event—because it is not necessary to build every sports infrastructure—is also positively associated with the support of hosting the OG. This is supported by the variable *Pollution*, which has a significant negative impact, indicating that the hosting is less supported when respondents think that the OG will increase pollution. However, the general level of concern regarding pollution resulting from the event is not high (on average, 2.82 on a scale from 1 to 5). People of RR might be already informed of the aim of hosting the OG in an economically and ecologically sustainable manner. It is further in line with recent research on large-scale events and their impact on pollution (37, 55). Contrary to initial concerns, Kim et al. (55) revealed that residents' perceptions of the negative impacts of traffic congestion and pollution during the 2002 FIFA World Cup were less severe than anticipated. Following the hosting of the G20 summit in Hangzhou, Li et al. (37) found a significant long-term improvement in urban air quality in Hangzhou.

Around 2/3 of those surveyed see the possible hosting of the event as an opportunity for Germany to present a different image of itself 100 years after the Nazi-Games in Berlin in 1936, which were misused by the Nazis for propaganda purposes. This is an important topic since former DOSB-President Alfons Hörmann mentioned concerns regarding Germany hosting 100 years after these Nazi-Games (42). Thomas Weikert, the current President of DOSB, expressed support for a potential bid in 2036 before acceding the role of DOSB-President. This notion is confirmed by the MLM. People who see the hosting 100 years after the Nazi-Games as a chance for Germany are more likely to support the potential hosting. This shows the historical significance that would arise from hosting of the OG 2036. This is particularly interesting as the 1972 Munich OG originally signaled the emergence of modern (West-) Germany (56). However, it seems that 50 years after the dawn of a new era in Germany, the topic resurfaced and the memory of Munich 1972 as a starting point diminished, especially as Munich 1972 is remembered primarily for the terrorist attacks on the Israeli team.

The average share of conservative voters was higher than the share of votes for the green party, which could be a simple explanation for the high level of support (12). However, the share of green votes is still much higher than in Wicker and Frick (12) (22.2% vs. 2.8%). Nevertheless, the three communities with the highest share of green votes (Aachen, Koeln, and Bonn) are among the four communities with the lowest average level of support (still >50%) for the potential hosting, and Gelsenkirchen, the community with the highest support by far (87.5%) is the community with the smallest amount of green votes (12.2%). This supports the assumption of Wicker and Frick (12) that green voters are rather negatively disposed toward the potential hosting. However, the average approval rating is still above 50%. As mentioned in the previous section, this could be explained by the fact that people are aware of the goal of sustainable OG, which could have led to green voters changing their minds in a more positive direction.

However, all structural variables in the MLM were not significant. This may imply that the structural differences between the 14 different cities are not too heterogeneous, suggesting a lack of variation at the contextual level. Consequently, individual factors may play a more decisive role. The non-significant findings on the structural level may also suggest that an MLM is not needed. However, the deviance, AIC, and BIC are much lower in the random intercept model compared to the null model. Consequently, the simultaneous estimation of individual and contextual characteristics using multilevel analysis is also empirically reasonable, and the estimation model is better suited to the data structure.

The results can be generalized to a certain extent, provided that the host region also has an existing infrastructure and a functioning democratic political system. The positive and negative influences of the Games should generally be communicated to the hosting population so that they can get a better picture of the advantages and disadvantages and decide for themselves whether an event is an option for them.

### 7.2 Limitations and future research

The limitations of the study should be pointed out, which at the same time opens research perspectives for further studies:

(i) At the contextual level, the 14 municipalities could be a rough and/or too aggregated measure of structural features/conditions because of the small sample size and because they may vary by districts in the municipalities. However, other studies also used small sample sizes at the contextual level in their MLMs (57, 58). Therefore, future research could build on our findings and incorporate other level structures of MLM with districts and/or the proximity of the respondents to the hosting venues, as Wicker and Frick (12) and Atkinson et al. (40) found. Moreover, the lack of significant findings on the structural level implies that further structural variables could be included in future research.
(ii) In addition, future research could include variables that further build on the rational choice model and are closely related to behavioral economics, such as a measure of political ideology. Trust in the host committee or political leadership is regularly used as a proxy in CVM research related to the hosting of major sporting events to control for a potential lack of trust in political institutions due to possible fears of corruption (9, 13).
(iii) A further limitation of our study is a potential selection bias in our voluntary survey that may inflate the level of support. However, the successful staging of the European Championships in Munich in August 2022 (after our survey) and the FISU World University Games in RR in 2025 could possibly have led (lead) to more support for the hosting of the OG 2036 in RR than our results suggest.

## 8 Conclusion

In November 2023, Andrea Milz (State Secretary for Sport and Volunteering in Northrhine-Westphalia) and Stephan Keller (Mayor of Duesseldorf) signed a letter of intent in which Northrhine-Westphalia officially declares its willingness to apply as a candidate for the German Olympic Sports Confederation's selection procedure to host the OG (59). The goal is to host an ecologically, economically, and socially sustainable event. The findings of this study address these points in the form of the negative impact of pollution and the positive impact of being a sustainable host, hosting 100 years after the Nazi-Games, and making the host population proud and thus creating intangible benefits. This should therefore be communicated to the public in order to increase public support for a possible bid by RR to host the OG in 2036 or later.

## Data Availability

The raw data supporting the conclusions of this article will be made available by the authors, without undue reservation.

## Footnotes

^1^The Olympic Agenda 2020 (OG20) is a strategic roadmap for the future of the Olympic Movement, presented by the International Olympic Committee (IOC) in December 2014. It contains a series of 40 recommendations aimed at ensuring the relevance, sustainability, and attractiveness of the Olympic Games. These recommendations include economic and environmental sustainability, credibility, and legacy.
